# Development and clinical validation of a seven-gene signature based on tumor stem cell-related genes to predict ovarian cancer prognosis

**DOI:** 10.1186/s13048-023-01326-8

**Published:** 2024-03-13

**Authors:** Guangwei Wang, Xiaofei Liu, Yue You, Silei Chen, Xiaohan Chang, Qing Yang

**Affiliations:** 1 Department of Obstetrics and Gynecology, Shengjing Hospital of China Medical University, Shenyang, China; 2 Shenyang Women’s and Children’s Hospital, Shenyang, China

**Keywords:** Tumor stem cell, Ovarian cancer, Marker, Prognosis

## Abstract

**Objective:**

Tumors are highly heterogeneous, and within their parenchyma, a small population of tumor-stem cells possessing differentiation potential, high oncogenicity, and self-renewal capabilities exists. These cells are pivotal in mediating tumor development, chemotherapy resistance, and recurrence. Ovarian cancer shares characteristics with tumor stem cells, making it imperative to investigate molecular markers associated with these cells.

**Methods:**

Stem cell-related genes were collected, and molecular subtypes were established based on gene expression profiles from The Cancer Genome Atlas using the R package tool “ConsensusClusterPlus.” Multi-gene prognostic markers were identified using LASSO regression analysis. Gene set enrichment analysis was employed to gain insights into the potential molecular mechanisms of these identified markers. The robustness of these prognostic markers was analyzed across different cohorts, and their clinical independence was determined through multivariate Cox analysis. A nomogram was constructed to assess the model’s clinical applicability. Immunohistochemistry was performed to validate the expression of hub genes.

**Results:**

Utilizing 49 tumor stem cell-related genes associated with prognosis, 362 ovarian cancer samples were divided into two distinct clusters, revealing significant prognostic disparities. A seven-gene signature (*GALP*, *CACNA1C*, *COL16A1*, *PENK*, *C4BPA*, *PSMA2*, and *CXCL9*), identified through LASSO regression, exhibited stability and robustness across various platforms. Multivariate Cox regression analysis confirmed the signature’s independence in predicting survival in patients with ovarian cancer. Furthermore, a nomogram combining the gene signature demonstrated strong predictive abilities. Immunohistochemistry results indicated significantly elevated *GALP*, *CACNA1C*, *COL16A1*, *PENK*, *C4BPA*, *PSMA2*, and *CXCL9* expression in cancer tissues.

**Conclusion:**

The seven-gene signature holds promise as a valuable tool for decision-making and prognosis prediction in patients with ovarian cancer.

## Introduction

Ovarian cancer is one of the most prevalent malignancies affecting the female reproductive system. In 2020, the United States anticipated 21,750 new cases of ovarian cancer and 13,940 ovarian cancer-related deaths [1]. Approximately 70% of patients with ovarian cancer are diagnosed at an advanced stage, primarily attributed to the asymptomatic nature of early-stage ovarian cancer and the absence of specific diagnostic methods [2]. The 5-year survival rates for stage III and stage IV ovarian cancer are < 40% and < 20% [3], respectively.

Tumor stem cells are subpopulations capable of self-renewal and differentiation into different tumor cell subtypes. They substantially influence tumor proliferation, metastasis, recurrence, and chemotherapy resistance [4–7]. In 2005, Bapat primarily identified a tumor-causing clone in malignant ascites from a patient with ovarian cancer using a multilayer sphere culture, offering evidential support for the existence of ovarian tumor stem cells [8]. Tumor stem cell-related genes exhibit significant expression within both ovarian surface epithelium and fallopian tube epithelium, potentially serving as the root cause of ovarian carcinogenesis [9]. Moreover, specific markers on the surface of ovarian cancer stem cells are closely linked to unlimited proliferation, infiltration, metastasis, drug resistance, and tumor recurrence [10, 11]. For instance, CD133 + ovarian cancer cells demonstrate heightened clonogenic and proliferative potential than CD133- cells, while elevated CD44 expression closely correlates with a poor prognosis of plasma ovarian cancer [12]. Therefore, exploring tumor stem cell-related genes is paramount to early diagnosis and targeted therapies for ovarian cancer.

More studies have emerged in recent years that integrate genomic data with bioinformatics analysis to prognosticate gynecologic malignancies [13]. Yang et al. constructed an 18 long-coding RNA (lncRNA) prognostic model for ovarian cancer based on ferroptosis-related lncRNAs [14]. Hu et al. established a five-gene signature from the RGS gene family to predict the ovarian cancer prognosis [15]. However, a critical research gap remains, as no studies have ventured into the classification of ovarian cancer or the prediction of ovarian cancer prognosis through the utilization of tumor stem-cell-related genes.

## Methods and materials

### Data sources and downloads

Gene expression profiling and clinical follow-up data were derived from The Cancer Genome Atlas (TCGA) database, utilizing RNA sequencing (RNA-Seq) data. The TCGA Genomic Data Commons application programming interface was employed to retrieve the latest clinical follow-up information, comprising 362 RNA-Seq data samples. The GSE32062 and GSE26193 datasets were acquired from the National Center for Biotechnology Information as validation cohorts. The GSE32062 and GSE26193 datasets featured 260 and 107 samples with clinical characteristics, respectively. The Molecular Signature Database V7.0 and Gene Ontology (GO) knowledgebase were used to identify human tumor stem-cell-related genes. A total of 456 genes associated with 30 tumor stem cell-related pathways were identified (Table 1).

**Table 1:** Pathways related to cancer stem cells in Reactome and GO databases

Stem cell function related pathways	PathwayID	Gene Count
GO:Somatic Stem Cell Population Maintenance	GO:0035019	72
GO:Negative Regulation Of Stem Cell Differentiation	GO:2,000,737	20
GO:Stem Cell Proliferation	GO:0072089	118
GO:Hematopoietic Stem Cell Differentiation	GO:0060218	79
GO:Negative Regulation Of Stem Cell Proliferation	GO:2,000,647	16
GO:Stem Cell Division	GO:0017145	41
GO:Hematopoietic Stem Cell Proliferation	GO:0071425	23
GO:Positive Regulation Of Stem Cell Differentiation	GO:2,000,738	20
GO:Regulation Of Stem Cell Population Maintenance	GO:2,000,036	28
GO:Neuronal Stem Cell Population Maintenance	GO:0097150	22
GO:Regulation Of Stem Cell Proliferation	GO:0072091	67
GO:Somatic Stem Cell Division	GO:0048103	24
GO:Stem Cell Differentiation	GO:0048863	248
GO:Positive Regulation Of Stem Cell Proliferation	GO:2,000,648	40
GO:Regulation Of Stem Cell Differentiation	GO:2,000,736	112
GO:Hematopoietic Stem Cell Migration	GO:0035701	6
GO:Stem Cell Fate Commitment	GO:0048865	9
GO:Mesenchymal Stem Cell Maintenance Involved In Nephron Morphogenesis	GO:0072038	6
GO:Mesenchymal Stem Cell Differentiation	GO:0072497	8
GO:Mesenchymal Stem Cell Proliferation	GO:0097168	5
GO:Asymmetric Stem Cell Division	GO:0098722	10
GO:egulation Of Hematopoietic Stem Cell Proliferation	GO:1,902,033	9
GO:ositive Regulation Of Hematopoietic Stem Cell Proliferation	GO:1,902,035	5
GO:egative Regulation Of Stem Cell Population Maintenance	GO:1,902,455	8
GO:ositive Regulation Of Stem Cell Population Maintenance	GO:1,902,459	8
GO:egulation Of Somatic Stem Cell Population Maintenance	GO:1,904,672	7
GO:Negative Regulation Of Somatic Stem Cell Population Maintenance	GO:1,904,673	5
GO:Regulation Of Stem Cell Division	GO:2,000,035	10
GO:Regulation Of Mesenchymal Stem Cell Differentiation	GO:2,000,739	6
Reactome Transcriptional Regulation Of Pluripotent Stem Cells	R-HSA-452723	31

### Data preprocessing

The TCGA RNA-Seq data were preprocessed as follows: 1) the samples lacking clinical data or exhibiting overall survival (OS) < 30 days were excluded; 2) the normal tissue sample data were removed; 3) the gene expression profiles relevant to stem cells were retained. The GSE32062 and GSE26193 datasets were preprocessed as follows: 1) the normal tissue sample data were eliminated; 2) the samples lacking clinical data or OS < 30 days were removed; 3) microarray probes were mapped to the human gene “SYMBOL” using the "Bioconductor package.” Detailed statistics of the preprocessed datasets are presented in Table 2.

**Table 2:** Clinical information of different cohorts

Characteristic		Training Set (*n* = 272)	Validation Set (*n* = 90)	*p* value	GSE32062 (*n* = 260)	GSE26193 (*n* = 107)
**Age(years)**	< 60	136	54	0.127	-	-
	≥ 60	136	36		-	-
**Survival Status**	Living	96	45	0.018	139	31
	Dead	176	45		121	76
**Grade**	G 1	1	0	0.426	0	7
	G 2	29	13		131	33
	G 3	235	74		129	67
	G 4	1	0		0	0
**Tumor Stage**	Stage I	1	0	0.383	0	21
	Stage II	15	5		0	10
	Stage III	218	64		204	59
	Stage IV	37	17		56	17

### Consistency clustering

The expression matrix of stem cell-related genes was extracted from TCGA data. The optimal number of clusters was determined based on the cumulative distribution function (CDF). Principal component analysis was applied to elucidate cluster differences and construct scatter plots.

### KEGG and GO enrichment analyses

Differentially expressed genes (DEGs) between Clusters 1 and 2 were calculated using Differential expression analysis of RNA-Seq data using the negative binomial distribution, version 2 (DESeq2). The DEGs were identified based on a false discovery rate (FDR) of < 0.05 and |log2FC|> 2 filtration criteria; volcano plots and heatmaps were generated to visualize these findings. The DEGs were subsequently enriched with the Kyoto Encyclopedia of Genes and Genomes (KEGG) and GO functions using the “clusterProfiler” R package.

### Protein interaction network and topological properties

The Search Tool for the Retrieval of Interacting Genes/Proteins (STRING) database (https://string-db.org/) [16] provides a comprehensive protein interaction network. Stem-cell-related genes were matched to the STRING database to elucidate the relationships between these DEGs, and interactions with scores > 0.7 were visualized using Cytoscape. Hub nodes were identified through Cytoscape’s “cytoHubba” module, and network topology was explored.

### Construction and evaluation of the predictive model for tumor stem cell-related genes

The 362 TCGA samples were randomly divided into groups (with a training cohort: validation cohort ratio of 3:1) to develop a gene signature for prognosis prediction. Only patients with an OS > 1 month were included in the survival analysis. To mitigate potential bias from a random distribution, 100 repeated samplings with replacement were performed across all samples in advance. The training and validation cohort samples were distributed based on age, stage, and grade. In the training cohort, univariate Cox regression analysis was performed using the “survfit coxph” function, with log-rank *p* < 0.05 as the threshold for identifying DEGs related to prognosis. We employed LASSO regression analysis to select tumor stem cell DEGs significantly associated with ovarian cancer prognosis, utilizing tenfold cross-validation and multivariate Cox regression analysis to determine the optimal genetic composition in the training cohort. The best lambda parameter and corresponding coefficient were chosen in the regression analysis using the “glmnet” R package. A seven-gene signature was constructed using the TCGA training set for prognosis prediction.

### Nomogram construction and evaluation

Univariate and multivariate Cox regression analyses assessed whether the seven-gene prognostic model remained independent of traditional clinical features. The coefficients obtained from the multivariate Cox regression model were utilized, and the “rms” R package was used to construct a nomogram. Furthermore, the “rmda” R package was used to generate a decline curve analysis (DCA) curve, while the “timeROC” R package was used to validate the superiority of the nomogram.

### Immunohistochemical staining evaluation

Tissue microarrays (HOvaC070PT01) comprising 65 ovarian cancer and five healthy ovarian tissue samples were purchased from Shanghai Outdo Biotech Co., Ltd. (Shanghai, China) to validate the expression of the seven genes in the signature. The studies adhered to the International Ethical Guidelines for Biomedical Research Involving Human Subjects (CIOMS), and the research protocols were approved by the Clinical Research Ethics Committee of Shengjing Hospital of China Medical University. The tissue microarray (TMA) slides were dried overnight at 37 °C, dewaxed in xylene, and rehydrated using graded ethanol. Subsequently, the tissue sections underwent microwave-based antigen retrieval in Ethylenediaminetetraacetic acid (EDTA) antigen repair buffer (pH 9.0). They were then treated with 3% hydrogen peroxide for 25 min to block endogenous peroxidase activity. The tissue was coated with 3% bovine serum albumin (BSA) and incubated at room temperature for 30 min to minimize non-specific reactions. Subsequently, the TMA slides were incubated with anti-C4BPA antibody (1:50 dilution; LifeSpan Biosciences, LS-C253165), GALP antibody (1:30 dilution; Sigma, HPA053938), CACNA1C antibody (1:200 dilution; Proteintech, 21,774–1-AP), PENK antibody (1:50 dilution; Sigma, HPA013138), PSMA2 antibody (1:100 dilution; Proteintech,14,377–1-AP), CXCL9 antibody (1:50 dilution; Proteintech, 22,355–1-AP), COL16A1 antibody (1: 50 dilution; LifeSpan Biosciences, LS-C198822), and left overnight at 4 °C. The tissues were then rinsed with 0.01 mol/L phosphate buffer saline (PBS) for 5 min each time. The tissues were incubated at room temperature for 50 min with the secondary anti-horseradish peroxidase (HRP) (labeled goat anti-rabbit, 1:200 dilution, Servicebio, GB23303). The sections were stained with 3,3-diaminobenzidine (DAB) after a PBS wash. Finally, the sections were counterstained with Mayer's hematoxylin, dehydrated, and fixed. To assess IHC staining, a semi-quantitative scoring criterion was applied [17]. The stained sections were scored by three pathologists blinded to the patients’ clinical characteristics. The scoring system is based on the proportion of positive cells in all tissue cells and the staining intensity of positive cells. Staining intensity is classified as follows: 0 (negative), 1 (weak), 2 (moderate), or 3 (strong). The staining ratio of positive cells is classified as: 0 (0 to 5%), 1 (6% to 25%), 2 (26% to 50%), 3 (51% to 75%), or 4 (> 75%). Based on staining intensity and the proportion of positive cells, the immunohistochemical results were categorized as follows: 0–1, negative (-); > 1–4, weakly positive ( +); > 4–8, moderately positive (+ +), and > 8–12, strongly positive (+ +  + +).

## Results

### Genotyping of ovarian cancer based on tumor stem cell genes

**Fig. 1:**
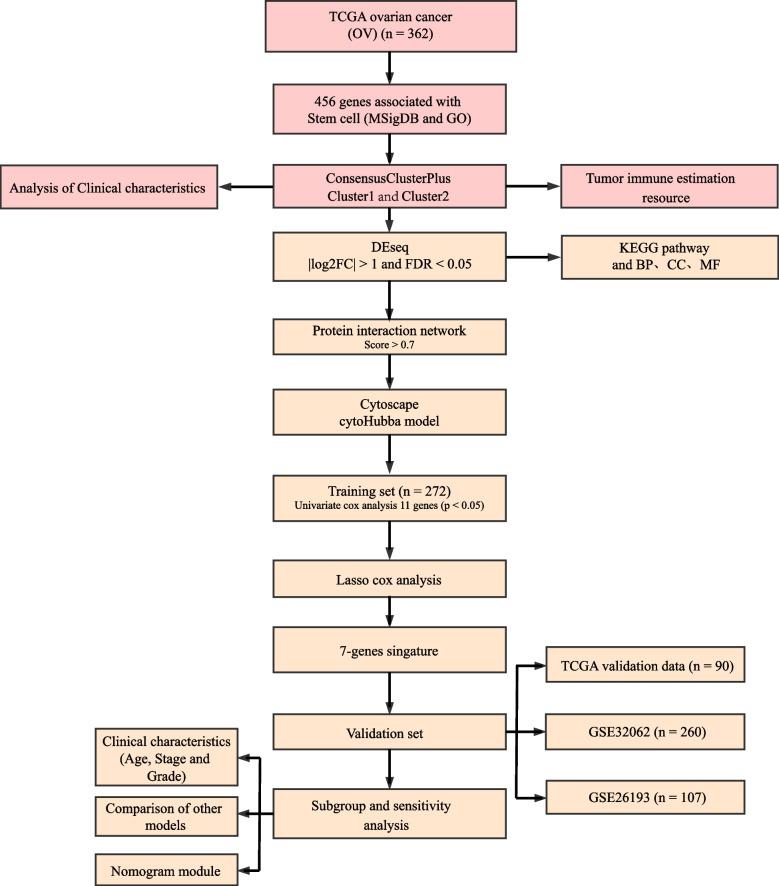
Flowchart

**Fig. 2:**
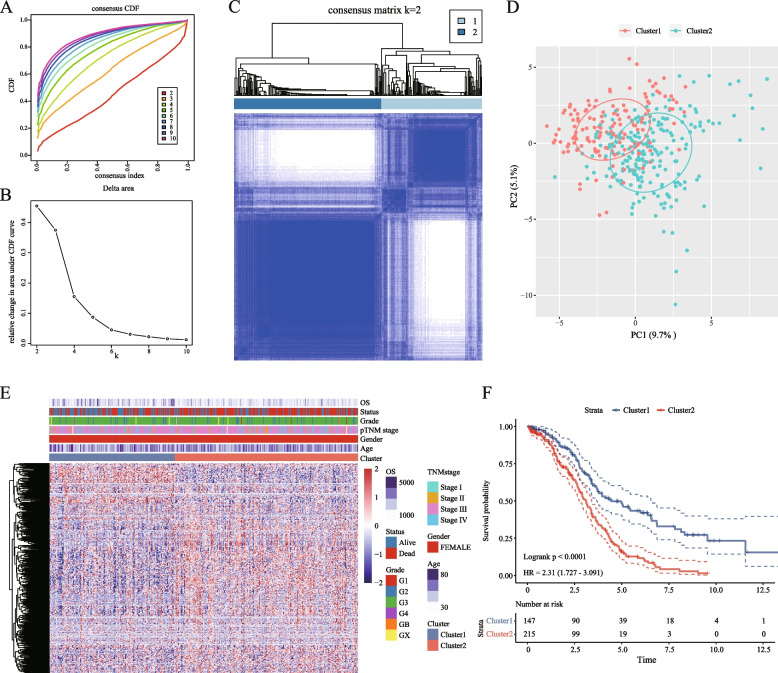
**A** Cumulative Distribution Function (CDF); **B** Delta Area Curve of consensus clustering, illustrating the relative change in the area under the CDF curve for each category number K compared with K-1; **C** Consensus K = 2; **D** Scatter plot of gene expression profiles in stem cells using principal component analysis (PCA); **E** Heatmap depicting stem cell gene expression; **F** Kaplan–Meier curve illustrating the prognosis of two clusters

A study flowchart for this article is shown in Fig. 1. The gene expression matrix of 428 tumor stem-cell genes was extracted from TCGA data, and 49 genes related to ovarian cancer prognosis (*p* < 0.05) were identified through univariate Cox analysis using the “coxph” function in R. The optimal cluster number was determined using CDF, and the CDF delta-area curve indicated that the two clusters produced the most stable results (Fig. 2A, B). Consequently, K = 2 was chosen, resulting in two molecular sets. The clustering results are shown in Fig. 2C. A total of 362 samples were assigned to these two clusters. Principal component analysis was performed on 428 stem cell gene sets, yielding the first two primary components and a corresponding scatter plot (Fig. 2D). Furthermore, a heatmap of these genes was generated (Fig. 2E), revealing distinct boundaries and prominent expression patterns for these genes within the two clusters. Kaplan–Meier analysis was employed to analyze prognosis differences (Fig. 2F), indicating that Cluster 2 had the worst prognosis.

### Comparison of clinical and immunological characteristics of molecular clusters

**Fig. 3:**
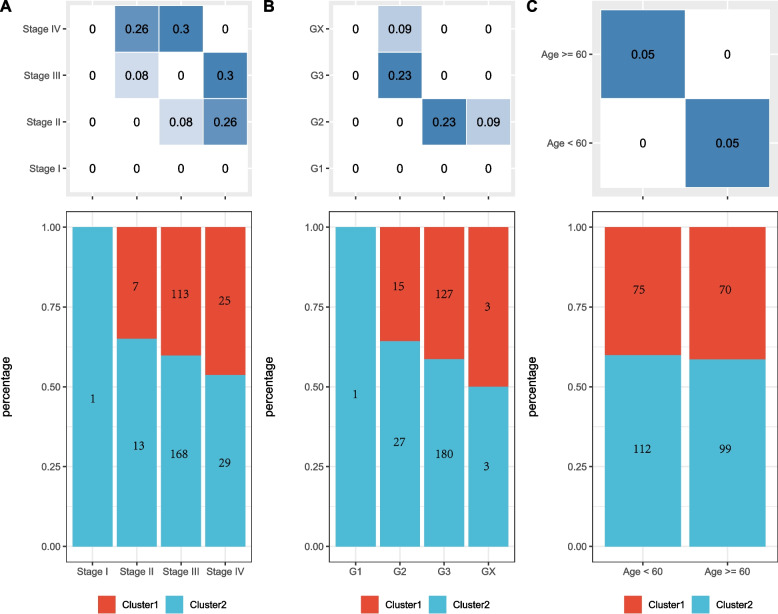
**A** Distribution of stage samples in two subgroups; **B** Distribution of grade samples in two subgroups; **C** Distribution of age samples in two subgroups, with the upper table showing the chi-square test results for clinical information between different clusters

**Fig. 4:**
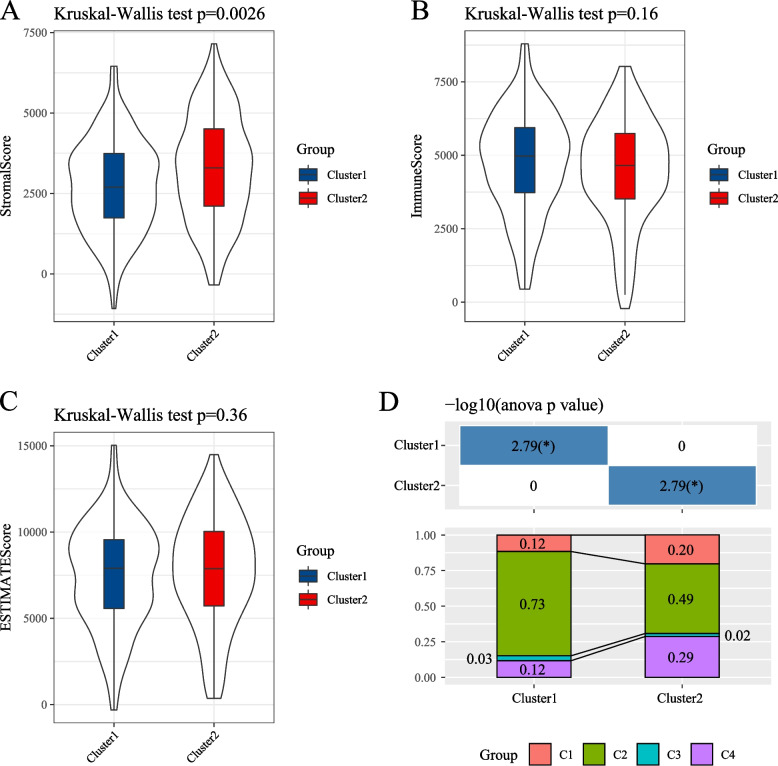
**A** ImmuneScore panel among molecular subtypes; **B** StromalScore panel among molecular subtypes; **C** EstimateScore panel among molecular subtypes; **D** Comparison of molecular subtypes with other subtypes, where different colors represent previously published subtypes

We further explored the relationship between ovarian cancer molecular clusters and clinical features based on tumor-associated stem cells. We compared the differences in clinical characteristics (including patient age, disease stage, and grade) between the two clusters. The results indicated differences between the clusters concerning Stage I and Grade I. However, the sample sizes for these two groups were small, as illustrated in Fig. 3. Furthermore, the immune characteristics of the molecular clusters were compared with those of existing subtypes. Each sample’s StromalScore, EstimateScore, and ImmuneScore were calculated using the “estimate” R package. The StromalScore of the Cluster 1 subtype was significantly lower than that of the Cluster 2 subtype (Fig. 4A-C). Among the 33 tumor types in TCGA, six immune subtypes were identified, including C1 (wound healing), C2 (INF-r dominance), C3 (inflammation), C4 (lymphocyte depletion), C5 (immunologically silenced), and C6 (TGF-β predominance) [18]. Cluster 1 contained significantly more C2 (INF-r dominance) than Cluster 2, whereas Cluster 2 contained significantly more C4 (lymphocyte depletion) and C1 (wound healing) than Cluster 1 (Fig. 4D).

### Identification and functional analysis of DEGs between clusters

**Fig. 5:**
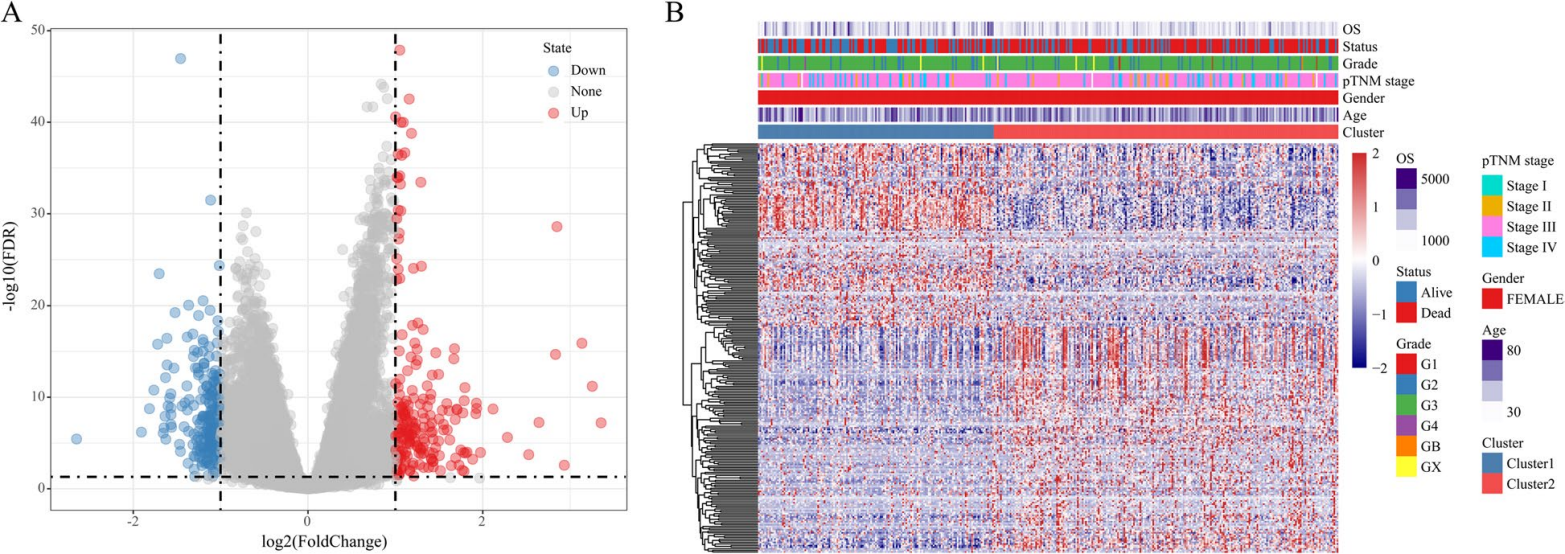
Volcano plot displaying differentially expressed genes (DEGs) between clusters 1 and 2; B: Heatmap of upregulated genes between the two clusters

**Fig. 6:**
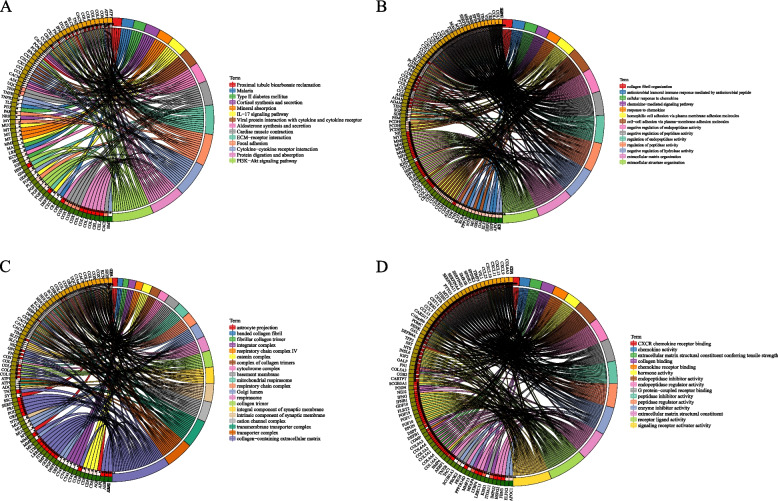
**A** Circular KEGG pathway enrichment map of DEGs; **B** Circular map illustrating biological process enrichment of DEGs; **C** Circular representation of the cellular component enrichment for DEGs; **D** Circular map displaying molecular function enrichment of DEGs, with different colors representing different pathways and connections denoting gene-pathway relationships

The DEGs between the Cluster 1 and Cluster 2 were identified using DESeq2. A total of 413 genes were obtained by filtering with FDR < 0.05 and |log2FC|> 2. Of these, 207 were upregulated, and 206 were downregulated. Volcano plots and heatmap representations of the DEGs in the two clusters are presented in Fig. 5A and B, respectively. The DEGs were further enriched in KEGG and GO pathways using “clusterProfiler,” with FDR < 0.05 as the threshold. The results revealed that the DEGs were enriched in 14 KEGG pathways (Fig. 6A), primarily the PI3K-Akt signaling and ECM-receptor interaction pathway. A total of 65 GO biological processes were enriched, including cell–cell adhesion via membrane adhesion molecules and adverse regulatory activity of hydrolases (Fig. 6B). Moreover, 20 GO cellular components were enriched, mainly the collagen-containing extracellular matrix, collagen trimer, and related cellular components (Fig. 6C). Finally, 39 GO molecular functions were enriched, primarily related to receptor-ligand activity and chemokine-receptor binding (Fig. 6D).

### Protein interaction network construction and analysis of topological properties

**Fig. 7:**
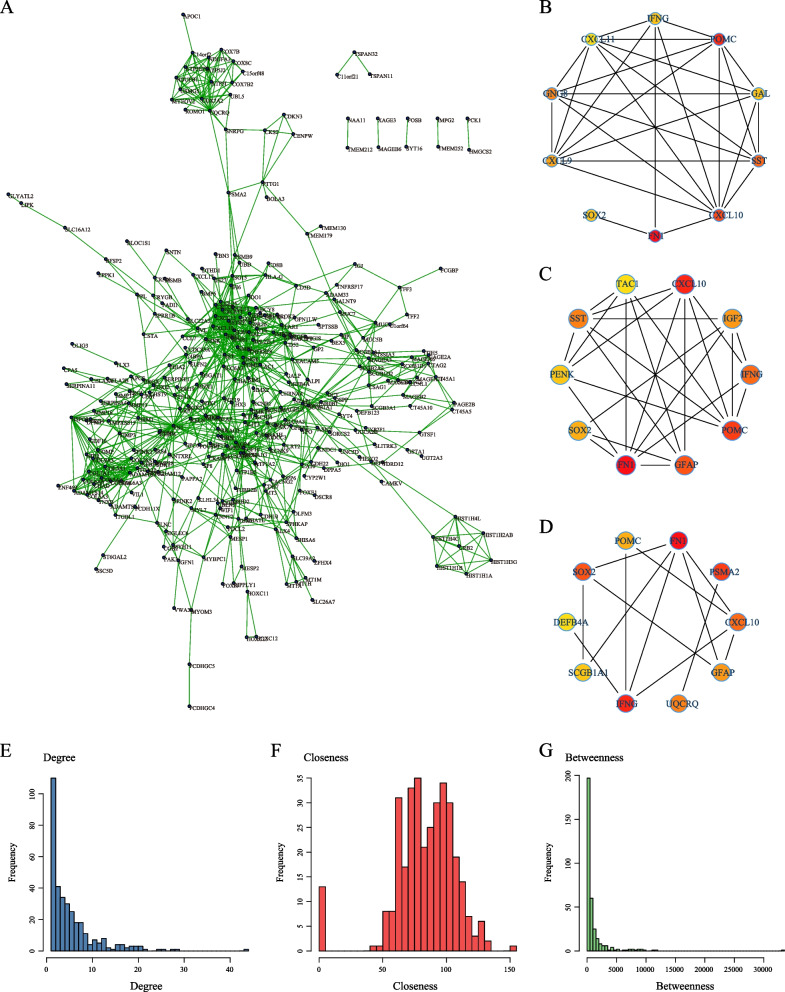
Protein–Protein Interaction (PPI) Network Analysis. **A** Mapping of 413 genes onto the PPI network; **B** Identifying hub nodes using the “degree” method; **C** Identifying hub nodes in the network using the “closeness” algorithm; **D** Identifying hub nodes using the “betweenness” algorithm, where a redder color indicates a higher score; **E** Degree distribution of the network; **F** Closeness distribution of the network; **G** Betweenness distribution of the network

The DEGs were mapped to the STRING database. Their interactions were obtained with scores > 0.7. These interactions were visualized using Cytoscape, resulting in 968 interactions among 413 co-expressed genes (Fig. 7A). The top 10 nodes were identified using the “cytoHubba” module in Cytoscape and calculated based on degree, closeness, and betweenness centrality methods (Fig. 7B-D). The results indicated that the hub genes identified using these three methods were consistent. When examining the network’s topological properties, the degree distribution followed a power law distribution (Fig. 7E), with most genes having degrees < 5. Moreover, the network’s closeness centrality analysis revealed that most nodes had relatively high closeness values, typically > 50 (Fig. 7F). The betweenness centrality analysis revealed values < 10 for most nodes (Fig. 7G). Nodes with high degrees, closeness, or betweenness were considered significant. We selected nodes with degrees, closeness, and betweenness values exceeding their respective medians as hub genes within the network. We identified 99 closely related genes associated with ovarian cancer development, which could serve as potential prognostic markers.

### Construction of prognostic risk model based on co-expressed genes

**Fig. 8:**
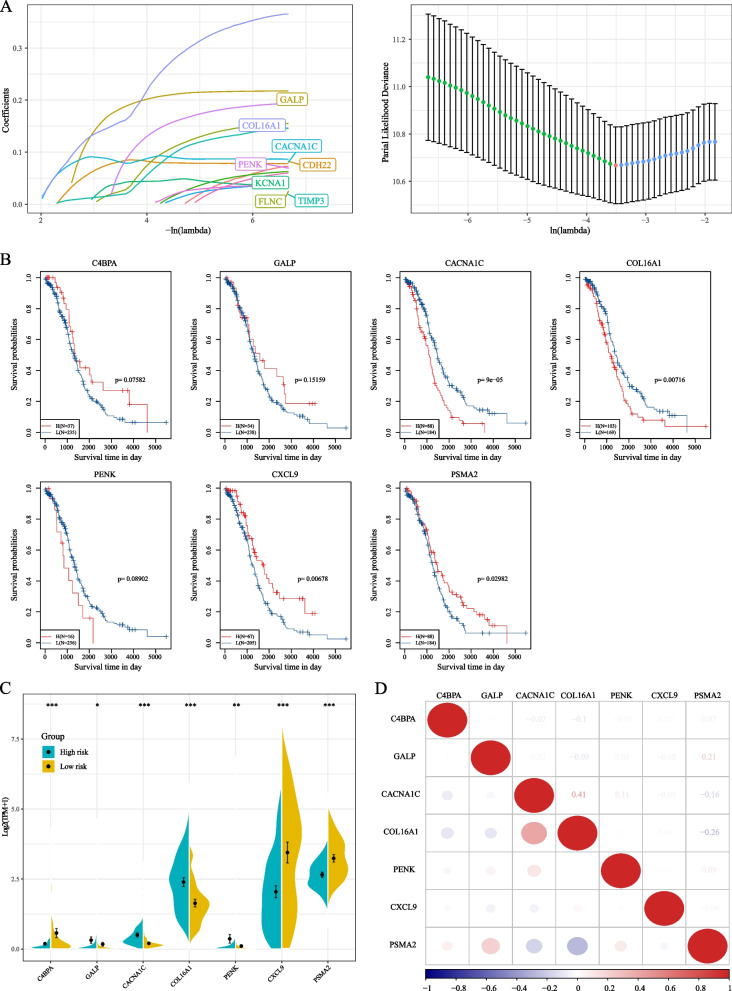
**A** Confidence intervals under each lambda; the trajectory of changes in each independent variable. The horizontal axis represents the natural logarithm (ln) value of the independent variable lambda, and the vertical axis represents the coefficient of the independent variable; **B** Kaplan–Meier (KM) survival curve for seven genes; **C** Distribution of expression levels of seven genes in the risk group; **D** Correlation heatmap of the seven-gene signature

**Fig. 9:**
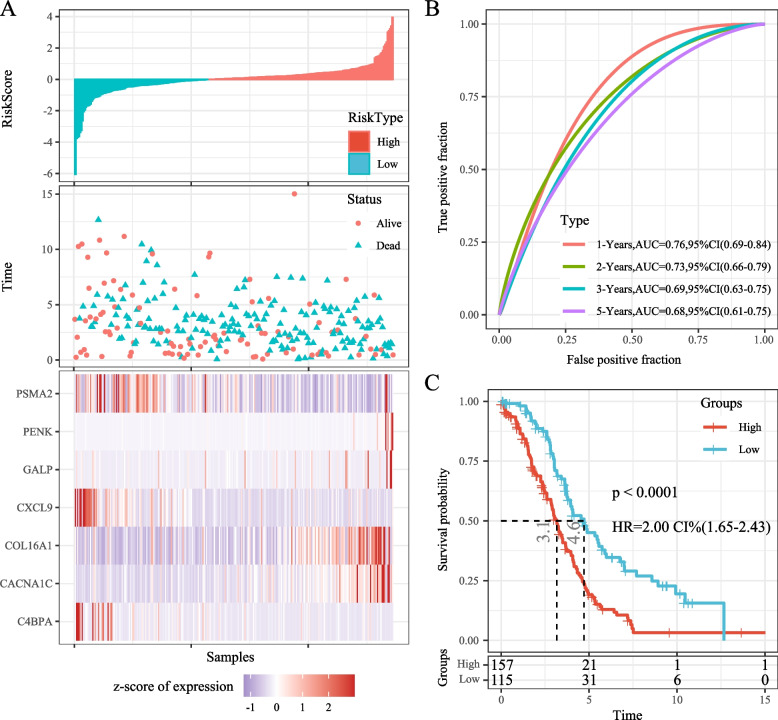
**A** Visualisation of risk scores, survival status, and expression levels of the seven genes in the training set; **B** Receiver Operating Characteristic (ROC) curve and area under the curve (AUC) for the classification based on the seven-gene signature; **C** KM survival curve distribution of the seven-gene signature in the training set

A univariate Cox proportional hazards regression model was applied to the initial pool of 99 candidate genes to build a prognostic risk model. This analysis identified 24 genes with significant prognostic differences. Subsequently, LASSO-Cox regression analysis was conducted using the “glmnet” package in R. The confidence interval under each lambda was analyzed (Fig. 8A); the model was optimal when lambda equaled 0.0299, leading to the selection of 12 genes as the target genes. Further refinement resulted in retaining seven of these genes (Akaike Information Criterion [AIC] = 1593.0) for the final model. Details regarding these seven definitive mRNA markers are provided in Table 3. The risk score formula is as follows: RiskScore7 = -0.313*C4BPA + 0.227*GALP + 0.116*CACNA1C + 0.212*COL16A1 + 0.184*PENK-0.412*CXCL9-0.145*PSMA2. Subsequent analysis revealed that CXCL9, PSMA2, CACNA1C, and COL16A1 could stratify patients into two groups with significantly different prognoses (*p* < 0.05) (Fig. 8B). There were significant differences in the expression levels of these seven genes between the high- and low-risk groups (Fig. 8C). Finally, a correlation analysis of these seven genes was conducted using the “corrplot” package in R (Fig. 8D). Calculating the risk score for each sample based on their gene expression levels, the distribution of risk scores among the samples was visualized (Fig. 9A). The results demonstrated that samples with high-risk scores exhibited significantly worse OS compared to those with low-risk scores. High expression of *GALP*, *CACNA1C*, *COL16A1*, and *PENK* were identified as risk factors, while increased expression of *C4BPA*, *PSMA2*, and *CXCL9* were recognized as protective factors. Furthermore, receiver operating characteristic (ROC) analysis was conducted to evaluate the prognostic classification of risk scores using the “timeROC” package in R. We assessed the predictive efficiency for prognosis at 1, 2, 3, and 5 years. The areas under the curve (AUCs) for 1 and 2 years were 0.76 and 0.73, respectively (Fig. 9B). Finally, we standardized the risk scores using z-score conversion and categorized samples with scores > 0 as the high-risk group and those with scores < 0 as the low-risk group. The Kaplan–Meier curve (Fig. 9C) displayed a significant difference between these groups (log-rank *p* < 0.0001, hazard ratio [HR] = 2.00).

**Table 3:** Multivariate cox analysis of 7-mRNA signature

Symbol	coef	HR	Low 95%CI	High 95%CI	P value
C4BPA	-0.313	0.731	0.561	0.953	0.021
GALP	0.227	1.254	1.091	1.443	0.002
CACNA1C	0.116	1.124	0.991	1.274	0.070
COL16A1	0.212	1.236	1.043	1.466	0.015
PENK	0.184	1.202	1.009	1.432	0.039
CXCL9	-0.412	0.662	0.499	0.879	0.004
PSMA2	-0.145	0.865	0.725	1.032	0.108

### Robustness verification

**Fig. 10:**
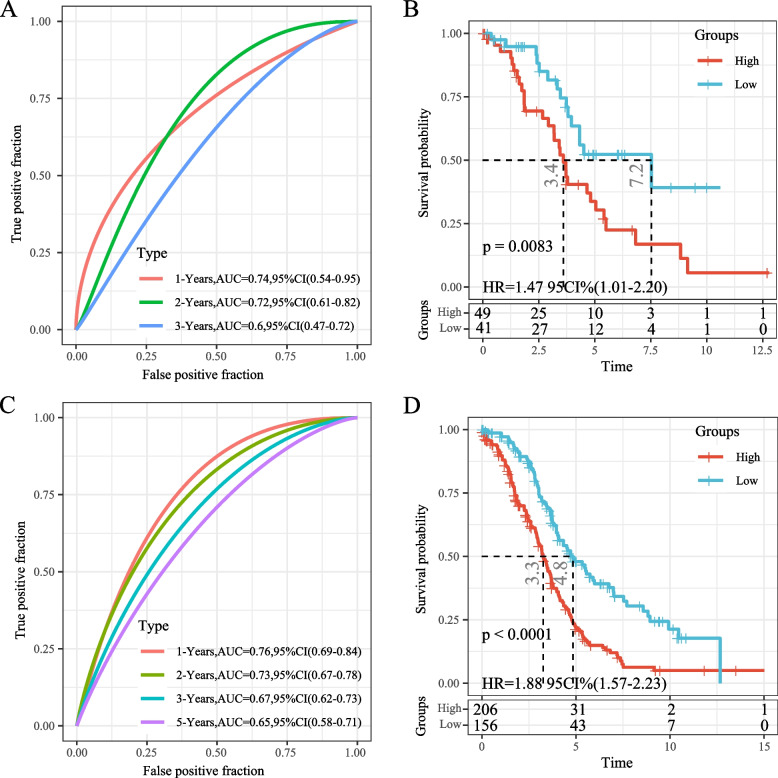
**A** ROC curve and AUC for the classification based on the seven-gene signature in the internal dataset; **B** KM curve distribution of seven-gene signature in the internal dataset; **C** ROC curve and AUC for the classification based on the seven-gene signature in the entire The Cancer Genome Atlas (TCGA) dataset; **D** KM curve distribution of the seven-gene signature in the entire TCGA dataset

**Fig. 11:**
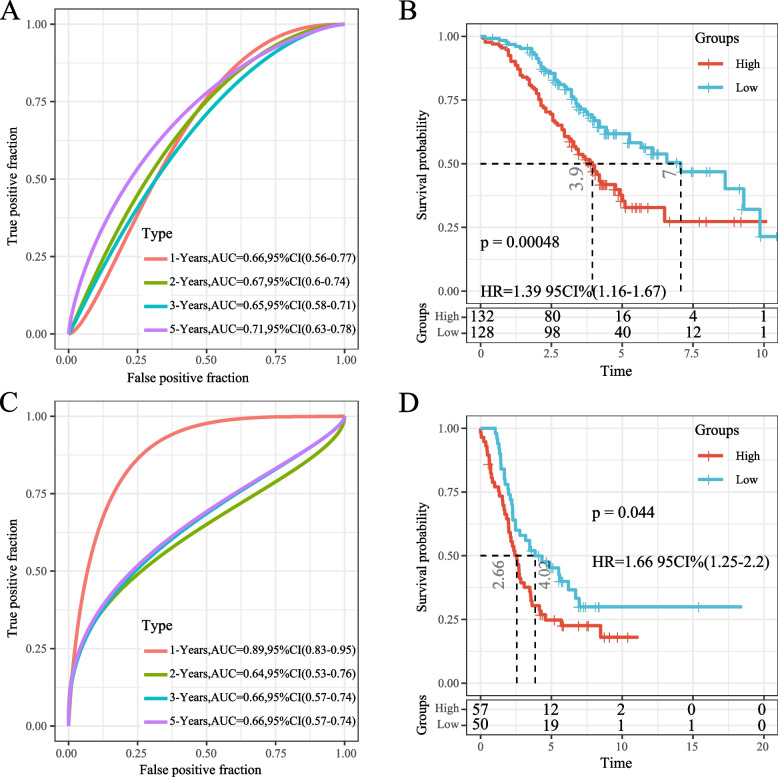
**A** ROC curve and AUC for the classification based on the seven-gene signature in the GSE32062 data set; **B** KM curve distribution of the seven-gene signature in the GSE32062 data set; **C** ROC curve and AUC for the classification based on the seven-gene signature in the GSE26193 dataset; **D** KM survival curve distribution of the seven-gene signature in the GSE26193 data set

The same methodology was used to analyze an internal validation dataset from TCGA database. The AUCs for predictive classification efficiency at 1 and 2 years were 0.74 and 0.72, respectively. There was a significant difference in the survival curves between the high- and low-risk groups (log-rank *p* = 0.0083, HR = 1.47) (Fig. 10A, B). Furthermore, all TCGA datasets were analyzed with the same model and coefficients, resulting in AUCs of 0.76 and 0.73 at 1 and 2 years, respectively. Significant differences in survival curves were observed between the high- and low-risk groups (log-rank *p* < 0.0001, HR = 1.88). Among the samples, 206 were classified as high-risk and 156 as low-risk (Fig. 10C, D). The same model and coefficients were used for external validation datasets GSE32062 and GSE26193. The 5-year AUC for GSE32062 was 0.71 (Fig. 11A), and the 1-year AUC for GSE26193 was 0.89 (Fig. 11C). Significant differences in survival curves were observed in both cohorts (Fig. 11B, D).

### Prognostic analysis and risk model evaluation

**Fig. 12:**
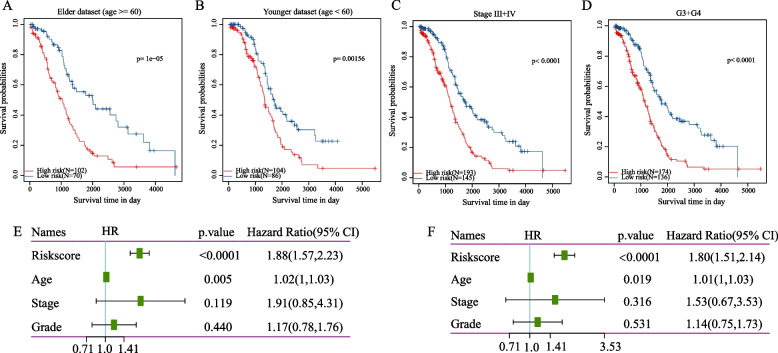
**A–D** Subgroup survival analysis based on Riskscore for different clinical cohorts, including younger, elder, Stage III + IV, and G3 + G4 patients. **E** Forest plot of univariate Cox analysis. **F** Forest plot of multivariate Cox analysis

Subgroup survival analysis showed that the seven-gene risk score effectively stratified patients based on age, stage (III + IV), and grade (G3 + G4) into high- and low-risk groups (Fig. 12A–D, *p* < 0.05). This finding indicated the model’s predictive capability across various clinical features. Multivariate Cox regression analysis revealed that the risk score was an independent prognostic risk for patients with ovarian cancer (HR = 1.80, 95% confidence interval [CI] = 1.51–2.14, *p* < 0.0001) (Fig. 12E–F), suggesting the seven-gene signature’s utility in clinical applications.

### Nomogram construction and evaluation

**Fig. 13:**
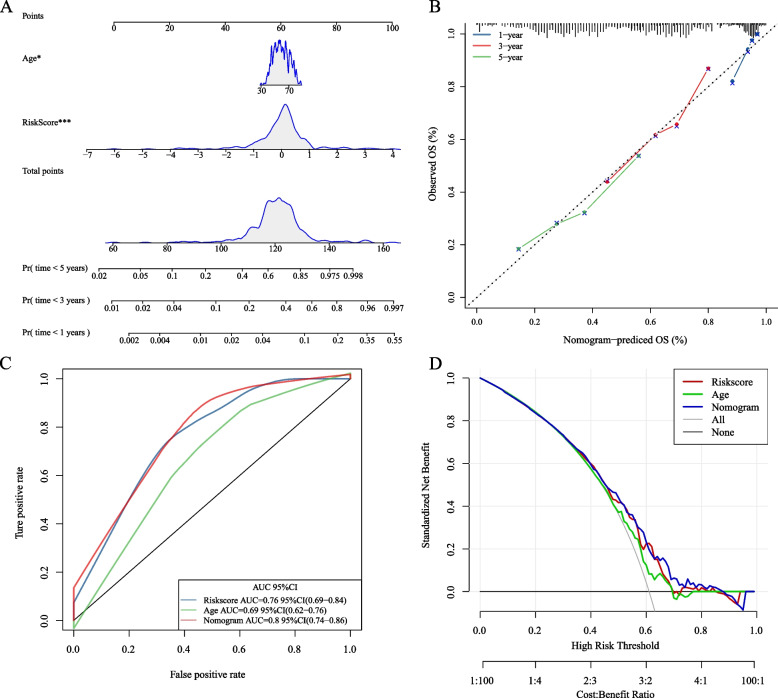
**A** Nomogram illustrating clinical variables and RiskScore. The nomogram calculates the probability of 1-year, 3-year, and 5-year OS by summing the points for each variable on the scale. **B** Calibration curve for predicting 1-year, 3-year, and 5-year OS in patients with HNSCC; **C** Time-dependent ROC curve analysis assessing the accuracy of the nomograms; **D** Decision curve analysis (DCA) curves intuitively evaluate the clinical benefit of the nomograms and their potential scope of application in obtaining clinical benefits. The calculated net benefits (Y-axis) are plotted against the threshold probabilities of patients having 5-year survival on the X-axis

A nomogram was constructed, incorporating age and the risk score. Each patient received a score for each prognostic parameter, with higher total scores indicating a worse prognosis (Fig. 13A). Furthermore, a calibration chart demonstrated that the 1-, 3-, and 5-year nomograms closely approximated the ideal model (Fig. 13B). The performance was assessed by comparing the AUCs of age, risk score, and nomogram using the “timeROC.” The nomogram exhibited a greater AUC than both the risk score and age (Fig. 13C). Finally, a DCA curve generated using the “rmda” package confirmed the superior predictive capabilities of our nomogram compared to the risk score and age (Fig. 13D).

### Risk model comparison with other models

**Fig. 14:**
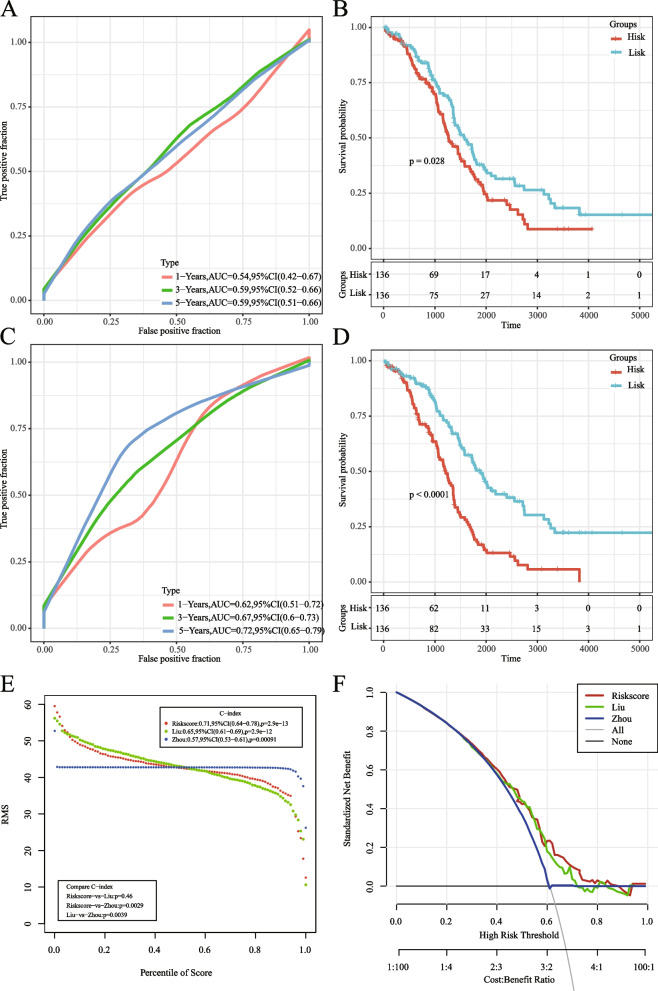
**A–B** AUC curve and prognostic KM curve for Zhou et al.'s model in TCGA dataset; **C–D** AUC curve and prognostic KM curve for Liu et al.'s model in TCGA data set; **E** Restricted mean survival (RMS) curves comparing the three models; **F** DCA curves for the three models

We compared our seven-gene model with two published prediction models: an 11-gene signature [19] and a three-gene signature [20]. To facilitate comparison, we calculated the risk score for each ovarian cancer sample in TCGA dataset using multivariate Cox analysis. We evaluated the ROC curves for each model and categorized samples into high- and low-risk groups based on the median risk score. ROC and Kaplan–Meier curves for the prognosis of the two models are presented in Fig. 14A–D. While the AUCs of the 11- and three-gene signatures were inferior to that of the seven-gene signature, significant differences in prognoses between high- and low-risk groups were observed for both models. Furthermore, we compared the restricted mean survival curve, demonstrating that our model exhibited the highest Concordance index (C-index) among the three (Fig. 14E). Clinical applicability was further assessed using DCA curves, which indicated the superior performance of our model compared to the others (Fig. 14F).

### Clinical validation of seven-gene expression

**Fig. 15:**
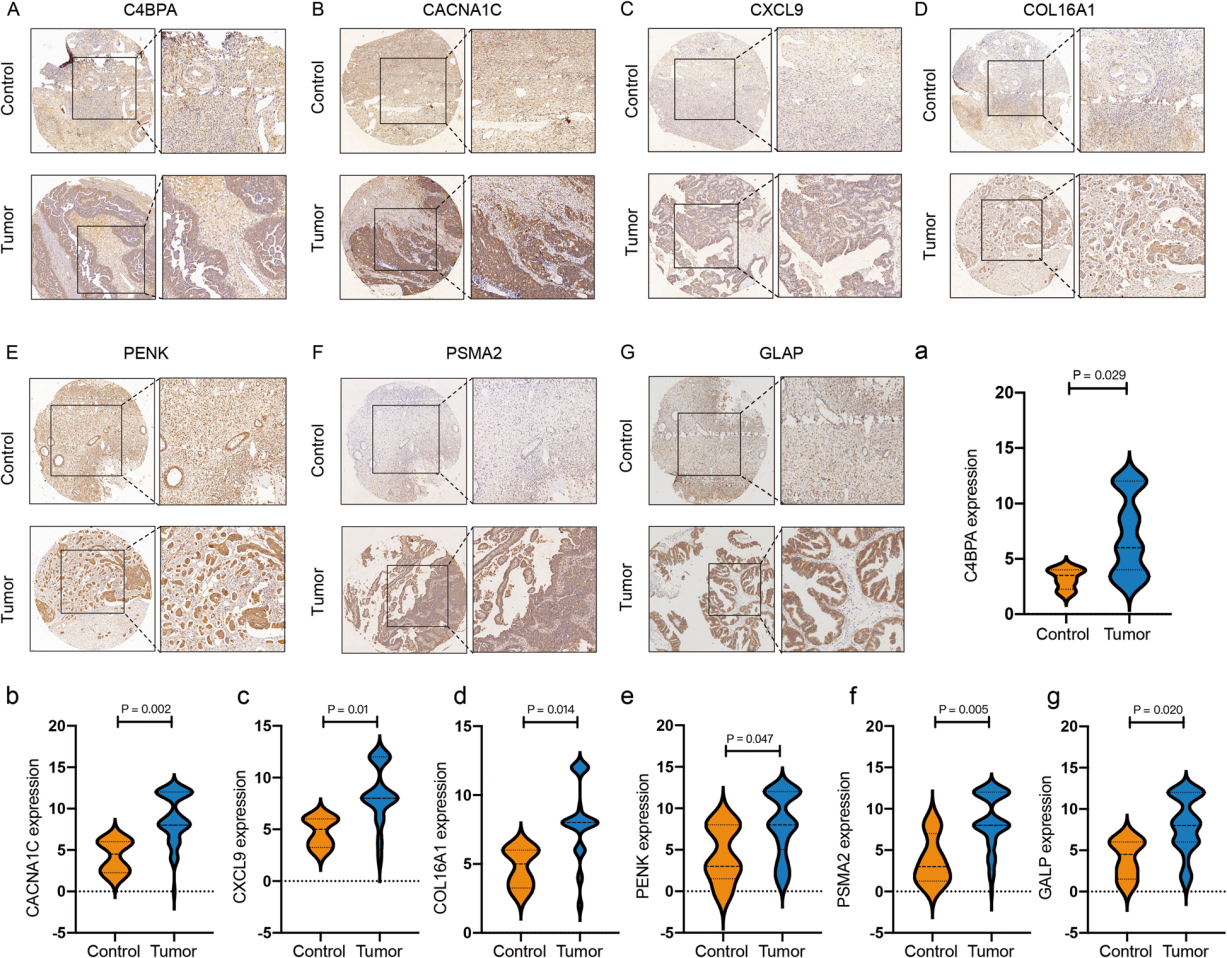
**A–G** Immunohistochemistry patterns depicting the expression of seven genes; a–g: Differential expression plots of the seven genes

To validate the expression of the seven hub genes, we analyzed 65 ovarian cancer samples and five healthy ovarian tissue samples. Immunohistochemistry results indicated significant upregulation of *GALP*, *CACNA1C*, *COL16A1*, *PENK*, *C4BPA*, *PSMA2*, and *CXCL9* in cancer tissues (Fig. 15A–G). Gene expression was visualized in immunohistochemistry using the “ggplot2” R package (Fig. 15a-g).

## Discussion

Ovarian cancer ranks as the seventh most prevalent malignancy in women and is the leading cause of death among gynecologic malignancies, contributing to 4% of cancer-related fatalities [21]. Recurrence and drug resistance are the principal factors underlying mortality in patients with advanced ovarian cancer, though the specific mechanisms remain elusive. The inherent heterogeneity within ovarian cancer contributes to disparate prognoses among patients with identical clinical stages, grades, and pathological types. Consequently, traditional prognostic methodologies frequently struggle to meet individualized needs, deliver accurate diagnoses, and identify optimal treatment options for ovarian cancer. Although numerous studies have employed gene expression profiles to stratify survival and prognosticate ovarian cancer across different cohorts, their clinical utility has been hindered by limited sample sizes and challenges in generating prognostic scores based on specific genes, thereby precluding their integration into clinical practice guidelines [22, 23].

Somatic mutations predominantly constitute cancer mutations, with approximately 90% of oncogenes displaying somatic mutations, 20% exhibiting germline mutations, and 10% manifesting common somatic and germline mutations [24]. Compared to germline mutations, somatic cell aberrations show more diverse patterns, including complex genome rearrangements. This divergence could be attributed to the relatively unconstrained evolutionary path of somatic cells, as mutations arising within subpopulations of cells could adapt for survival. In contrast, germline mutations are universally present in almost all cells throughout development. Cancer is frequently conceptualized as an evolutionary process characterized by genetic instability and natural selection, driven by the constant accumulation of somatic mutations [25]. The persistent somatic evolution occurring during tumor progression contributes significantly to genetic heterogeneity. Somatic cells could be reconverted into stem cells [26]; this hypothesis underscores a possible association between somatic cell mutations and cancer stem cells.

Tumor stem cells are central to treatment failure, metastasis, and recurrence due to their enhanced tumorigenicity and chemotherapy resistance. Tumor stem cells are essential in ovarian cancer’s recurrence, metastasis, and chemotherapy resistance [27, 28]. Targeting tumor stem cells emerges as an effective strategy to improve the prognosis of epithelial ovarian cancer [29]. In this study, we genotyped 362 ovarian cancer samples from TCGA database, focusing on 49 prognosis-associated tumor-stem-cell genes. These samples were subsequently categorized into two clusters, revealing significant differences in prognosis. Subsequently, a seven-gene signature model (including *GALP*, *CACNA1C*, *COL16A1*, *PENK*, *C4BPA*, *PSMA2*, and *CXCL9*) was constructed based on hub genes identified in a protein-interaction network; four of these genes were identified as risk factors, while three acted as protective factors. Utilizing this seven-gene signature, we effectively classified patients based on age, disease stage (III + IV), and grade (G3 + G4) into high- and low-risk groups, with the latter exhibiting a more favorable prognosis. Furthermore, the seven-gene signature demonstrated robust predictive abilities across various clinical features. When constructing predictive models, we compared the performance of age, risk score, and the seven-gene signature, with the combined approach yielding superior results. Our seven-gene signature prediction model exhibited enhanced performance compared to previous studies, likely attributable to our utilization of RNA-Seq data for model development and validation, in contrast to the data generated from different platforms in most prior research. Regarding the constituents of our 7-gene signature, *CACNA1C* is overexpressed in high-grade serous ovarian cancer and correlated with prognosis [30], whereas *COL16A1* expression is significantly correlated with progression-free survival in advanced serous ovarian cancer [31]. *CADM1* overexpression potentially inhibits the migration and invasion of ovarian cancer cells by regulating the upstream regulatory factor C4b-binding protein (C4BPA) and the PI3/Akt/mTOR signaling pathway [32]. *PSMA2* overexpression is observed in ovarian cancer [33], and chemokine ligand 9 (CXCL9) is a vascular inhibitor that could inhibit ovarian cancer through lymphocyte invasion [34, 35].

This study possesses certain limitations. Firstly, this retrospective analysis, which relies on public datasets, should be complemented by a prospective study with a larger sample size for further validation. Secondly, the highly heterogeneous nature of ovarian cancer might challenge the validity of our seven-gene signature due to potential sampling bias.

Additionally, our study faced limitations at the immunohistochemistry stage, as the absence of clinical prognostic information for these samples prevented us from establishing a direct relationship between gene expression and prognosis. Future research should explore the relationship between gene expression, related protein levels, and patient prognosis with available clinical prognostic data to enhance the accuracy of our model. Furthermore, the limited depth of research on these genes in ovarian cancer necessitates further investigation into their biological roles and mechanisms within the context of this disease.

In conclusion, ovarian cancer was classified into two stemness-related clusters utilizing tumor stem cell-related genes with distinct prognostic features and tumor microenvironment patterns. The seven-gene signature offers a promising tool for predicting the prognosis of patients with ovarian cancer and guiding clinical decision-making.

## Data Availability

The datasets used and/or analyzed during the current study are available with the corresponding author upon reasonable request.
